# Adaptations in the Structure and Function of the Cerebellum in Basketball Athletes

**DOI:** 10.3390/brainsci15111221

**Published:** 2025-11-13

**Authors:** Yapeng Qi, Yihan Wang, Wenxuan Fang, Xinwei Li, Jiaxin Du, Qichen Zhou, Jilan Ning, Bin Zhang, Xiaoxia Du

**Affiliations:** 1 School of Psychology, Shanghai University of Sport, 399 Changhai Road, Yangpu District, Shanghai 200438, China; 2211916004@sus.edu.cn (Y.Q.);; 2 School of Athletic Performance, Shanghai University of Sport, Shanghai 200438, China; 3 Centre for Advanced Imaging, The University of Queensland, St Lucia, Brisbane, QLD 4072, Australia; 4 Center for Exercise and Brain Science, Shanghai University of Sport, 399 Changhai Road, Yangpu District, Shanghai 200438, China

**Keywords:** cerebellum, basketball athlete, multimodal magnetic resonance imaging

## Abstract

**Background/Objectives**: The cerebellum contributes to both motor and cognitive functions. As basketball requires the integration of these abilities, basketball athletes provide an ideal model for exploring cerebellar adaptations. This study aimed to examine multidimensional cerebellar adaptations in basketball athletes and their associations with physical performance. **Methods**: In this study, 55 high-level basketball athletes and 55 non-athletes matched for age and gender were recruited for multimodal magnetic resonance imaging data collection and physical fitness tests. We compared the structural and functional differences in the brain between the two groups and analyzed the correlations between regional brain indices and physical fitness test outcomes. **Results**: Basketball athletes exhibited increased gray matter volume in Crus I, alongside heightened ALFF signal in Crus I and improved regional homogeneity in Crus II and VII b compared to non-athletes. Diffusion kurtosis imaging analysis demonstrated that athletes perform elevated kurtosis fractional anisotropy and decreased radial kurtosis within the cerebellar cortex and peduncles, with cortical modifications mainly localized around Crus I and lobule VI. Notably, both kurtosis fractional anisotropy and the amplitude of low-frequency fluctuations displayed positive correlations with vertical jump performance, an indicator specific to basketball ability. **Conclusions**: Basketball athletes exhibit structural, microstructural, and functional cerebellar adaptations, especially in Crus I. These modifications involve regions associated with motor and cognitive representations within the cerebellum, and part of the indexes are linked to the athletes’ physical performance. This study enhances our understanding of cerebellar adaptive changes in athletes, providing new insights for future research aimed at fully elucidating the role of the cerebellum in these individuals.

## 1. Introduction

The cerebellum is a key brain structure involved in both motor and various cognitive functions, such as motor control, executive control, and working memory [1,2,3]. It has been suggested that the cerebellum develops internal models to optimize motor and cognitive processes to facilitate behavior performance across different contexts [4]. The cerebellum can automatically maintain neural activity around a homeostatic baseline [5], and the enhancement or impairment of cerebellar regions can exert a wide impact on motor or cognitive functions [6]. Cerebellar modulation may increase the working efficiency of the cerebral cortex [7]. In-depth study of the cerebellum is of great significance for our comprehensive understanding of brain function [8,9].

Evidence from animal and human studies has shown that long-term motor training is associated with structural and functional cerebellar adaptations [10,11,12,13]. At the macrostructural level, athletes engaged in different types of sports display altered gray matter volume (GMV) in different cerebellar regions [11,14]. At the microstructural level, sports training has been linked to changes in axonal organization and synaptic remodeling [10,12,15], which can be captured by diffusion imaging indices [16]. Functionally, resting-state and task-based fMRI studies have revealed sport-specific modulations of spontaneous activity and regional coherence within the cerebellum [13,17]. Collectively, cerebellar adaptations emerge with long-term sport training, with the direction and magnitude of changes appearing to depend on the nature of the sport.

Studies on professional athletes further revealed the relationship between cerebellar adaptations and specific sport performance demands. For example, athletes’ neuromuscular performance in the agility test is associated with the GMV of the posterior cerebellar lobe [18]. Additionally, the functional and structural characteristics of the anterior cerebellar lobe are closely associated with athletes’ motor speed [19]. These observations suggest that cerebellar adaptations may be functionally meaningful and are potentially linked to athletes’ specific capabilities in their chosen sport. However, the relationship between cerebellar adaptation and specific sport performance remains incompletely understood.

Basketball, a typical open-skill sport, requires both refined motor execution and rapid cognitive processing under unpredictable game conditions, such as precise shooting and tactical decision-making [20]. These cognitive-motor integration demands closely align with the functional characteristics of the cerebellum, making basketball athletes a compelling model for examining cerebellar adaptations. Nevertheless, the structural and functional adaptations of the cerebellum in basketball athletes remain poorly understood. Based on this background, the present study employed multimodal MRI to investigate cerebellar adaptations in professional basketball athletes compared with healthy controls, focusing on three dimensions: macroscopic structure, resting-state function, and microstructure. In addition, both general and basketball-specific physical fitness were assessed to explore how cerebellar adaptations relate to athletic performance. To our knowledge, this is the first study to systematically characterize cerebellar adaptations in basketball athletes across multiple levels of neural organization, providing new insights into cerebellar function and its relationship with sport performance.

## 2. Materials and Methods

### 2.1. Participants

Participants were recruited via online advertisements and on-campus posters. The basic inclusion criteria were as follows: (1) aged 18–28 years, with a relatively narrow age range to control for developmental factors effectively; (2) normal or corrected-to-normal vision, to ensure the smooth progress of subsequent experiments; (3) right-handed, to control the influence of lateralization on brain structure and function results; (4) good physical health, with no implantable medical devices, to ensure the feasibility of MRI scanning; (5) normal cognitive function, with no history of traumatic brain injury or mental illness, to ensure the smooth conduct of the experiment and exclude other confounding factors.

Furthermore, participants in the athlete group were required to meet the following criteria: having a national second-level or above grade in basketball and having at least 5 years of specialized basketball training experience, to ensure the professionalism of sports skills and the richness of sports experience within the athlete group. Participants in the control group had to meet an additional requirement: no professional sports training experience and no consistent exercise habits lasting longer than one year to prevent interference from other sports training experiences.

All subjects who do not meet the aforementioned basic or group specific criteria will be excluded. For example, left-handed individuals will be excluded from both groups, and individuals with any professional sport experience will not be included in the control group. Ultimately, fifty-five basketball athletes (mean age: 22.75 ± 1.83 years) and fifty-five healthy non-athletes of the same age and gender (mean age: 22.87 ± 1.98 years) were recruited. Detailed information regarding the participants is provided in Table 1. All participants provided informed consent prior to participation.

### 2.2. Physical Abilities Test

In consultation with professional basketball coaches, we conducted a comprehensive evaluation of the athletes’ physical abilities to investigate their potential associations with cerebellar adaptations. Tests were structured along two dimensions. First, basketball-specific physical abilities were assessed to evaluate basketball-related motor skill level, including vertical jump, shooting accuracy, and dribbling. These three indicators represent the core sport-specific abilities of basketball athletes [21,22,23]. Second, general physical ability assessments were designed to determine whether the motor advantages of basketball athletes extend to non–sport-specific domains. These tests included agility, gait speed and explosive power, all of which are fundamental to daily movement.

All assessments were guided by a national-level basketball coach from Shanghai University of Sport and aligned with the guidelines from an authoritative book on physical abilities evaluation [24]. The assessments were administered by graduate students specializing in basketball to ensure the professionalism and validity of the testing process. Detailed testing and scoring methods for each test are provided in Supplementary Table S1.

### 2.3. MRI Acquisition

MRI data were acquired using a 3.0 Tesla Siemens Prisma scanner with a 64-channel head/neck coil. The scanning protocol sequentially comprised: (1) an 8-min resting-state BOLD weighted fMRI acquisition during which participants-maintained attention on a central “+” displayed against a black background while remaining alert and relaxed, (2) 6-min T1-weighted structural and (3) 8-min diffusion-weighted imaging (DWI) acquisitions. To minimize motion artifacts, participants were instructed to maintain head immobilization with foam padding throughout the scanning process.

To ensure the data quality, we also conducted a pre-scan preparation protocol: ≥6 h of sleep the night before, abstinence from alcohol/caffeine for 48 h, and avoidance of vigorous physical activity on scan day. Field map information was also acquired before the functional and diffusion scans to correct for image distortions caused by field inhomogeneity. Full imaging parameters are detailed in Table 2.

### 2.4. VBM Analysis

To better analyze the GMV of the cerebellum, T1-weighted structural images were preprocessed and analyzed with the SUIT toolbox (https://www.diedrichsenlab.org/imaging/suit.htm, accessed on 10 November 2025) in SPM12. After reorienting and resampling, structural images were automatically isolated, and spatial normalization was performed in the SUIT space [25] using the DARTEL algorithm. Finally, the isolated images were modulated and smoothed using a Gaussian kernel with a full width at half maximum of 2 mm. To ensure data quality, additional analyses were conducted on structural images using CAT12 (https://www.nitrc.org/projects/cat/, accessed on 10 November 2025). According to the results of the CAT12 output, two participants whose data quality was rated B- or lower were excluded. Additionally, this analysis provided each participant’s total intracranial volume (TIV), which was included as a covariate in subsequent statistical analyses to minimize the influence of absolute brain size on the results.

### 2.5. Resting-State fMRI Analyses

Resting-state fMRI analysis was performed using DPABI v8.0 on MATLAB 2022a (Natick, MA, USA) [26]. Since we introduced three dummy scans (totaling 6 s) at the beginning of the scans, no volumes were removed from the data. Each subject’s 240 functional images were realigned and unwrapped (using a field map), linearly coregistered with structural data, slice timing corrected, regressed for white matter and cerebrospinal fluid signals, smoothed using a Gaussian kernel of 4 mm full width at half maximum, and band-pass filtered with a frequency window of 0.01 to 0.1 Hz. We applied the 4-eye reliability principle to ensure data quality, where two independent researchers evaluated the imaging data. Based on this assessment, participants with poor data quality or head movements exceeding 2 mm of translation or 2 degrees of rotation were excluded, resulting in the exclusion of 7 non-athlete participants and 6 athlete participants from subsequent analysis.

Then, we calculated the Amplitude of Low-Frequency Fluctuations (ALFF), fractional ALFF (fALFF), Regional Homogeneity (ReHo), and Degree Centrality (DC) values within the whole brain of each participant in the original space based on the preprocessed resting-state data. ALFF and fALFF reflect spontaneous activity in individual voxels [27], while ReHo and DC reflect the correlation between individual voxels and other voxels [28]. The combined use of these indicators can provide a more comprehensive description of brain function changes. Notably, band-pass filtering was not performed before ALFF and fALFF calculations, and spatial smoothing was not performed before ReHo and DC calculations. We segmented these calculated results (original space) using the SUIT template [25]. We registered them to the SUIT space using the deformation file obtained from normalization in VBM analysis (described in above section).

Two-sample *t*-tests were then used to compare ALFF, fALFF, ReHo, DC, and GMV map differences between the basketball athlete and control groups. Results were thresholded at an uncorrected voxel *p* < 0.001 with an FDR-corrected cluster threshold < 0.05. TIV was introduced as a nuisance covariate in GMV analysis. The between group analysis was limited in the SUIT template mask.

### 2.6. Diffusion Kurtosis Image Analysis

Diffusion data were preprocessed and analyzed using FSL (https://fsl.fmrib.ox.ac.uk/fsl/docs/index.html, accessed on 10 November 2025), MRtrix3 [29], and DKE software (https://www.nitrc.org/projects/dke/, accessed on 10 November 2025). Processing steps followed a previously described pipeline [30], including noise correction [31], Gibbs ringing correction [32], susceptibility-induced distortions, head motion correction, outlier replacement, and eddy-current-induced distortions corrections using field map (https://fsl.fmrib.ox.ac.uk/fsl/docs/registration/fugue.html, accessed on 10 November 2025) and eddy (https://fsl.fmrib.ox.ac.uk/fsl/docs/diffusion/eddy/index.html, accessed on 10 November 2025), and n4 bias field correction. Employing the processed multi-shell data, diffusion kurtosis image (DKI) metrics were estimated by jointly using of all shells’ data in DKE software, including Diffusion Tensor Image (DTI) parameters: fractional anisotropy (FA), radial diffusivity (RD), axial diffusivity (AD), mean diffusivity (MD), and DKI parameters: kurtosis FA (KFA), mean kurtosis (MK), axial kurtosis (AK), and radial kurtosis (RK) [33]. See Supplementary Table S2 for definitions of DTI and DKI parameters.

The standard FSL pipeline for Tract-Based Spatial Statistics (TBSS) was applied to state DTI and DKI metric maps. Based on our visual inspection, following the 4-eye reliability principle, 5 participants were excluded from the statistical analysis: 2 from the control group and 3 from the athlete group. We analyzed inter-group differences using a permutation test with 5000 iterations, corrected by threshold-free cluster enhancement (TFCE) at *p* < 0.05, and restricted to a whole brain mask.

### 2.7. Correlation Analysis

Based on the surviving clusters identified in between-group analysis, we extracted athletes’ individual values of GMV, KFA, RK, ReHo (z-scores), and ALFF (z-scores) for correlation analysis with demographic and physical performance measures. These measures included age, years of education, years of training, years of basketball training, training time, shooting percentage, vertical jump, dribbling, agility, gait speed, and explosive power. Since some variables deviated from normality, Spearman correlation analysis was applied, with statistical significance determined using a threshold of *p* < 0.05 (FDR corrected). All analyses were conducted in R 4.4.0 using the “BruceR” package 2024.6. For comparative purposes, we also performed Pearson correlation analysis while controlling for gender, age, and years of education. The results showed no substantial differences between the two correlation methods (see Tables S3 and S4 for details).

## 3. Results

### 3.1. Demographic and Physical Data

The demographic and physical data of the athlete and control groups are presented in Table 1. Demographic factors did not differ significantly between the basketball athletes and controls and remained balanced after exclusion in all analyses. Basketball athletes demonstrated significantly better agility than non-athletes (T = 6.43, *p* < 0.001) in terms of general physical abilities.

### 3.2. Grey Matter Volume

**Figure 1:**
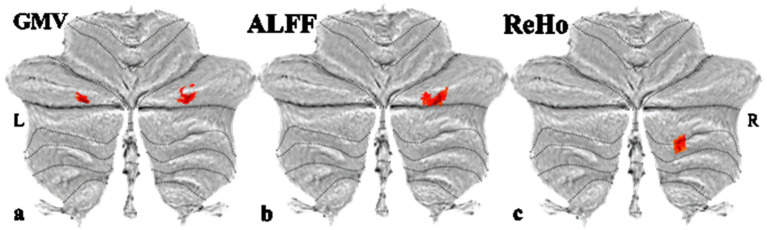
Significant differences in GMV, ALFF, and ReHo between basketball athletes and non-athletes (FDR-corrected *p* < 0.05). (**a**) Compared with non-athletes, the athletes showed significantly greater GMV in the bilateral cerebellar Crus I. (**b**) The athletes exhibited significantly higher ALFF values in the right cerebellar Crus I than non-athletes. (**c**) Compared with non-athletes, the athletes showed significant higher ReHo values in the right cerebellar crus II to lobule VII b. Red indicates higher values in athletes compared to the control group.

Athletes exhibited significantly greater GMV in the bilateral cerebellar Crus I (Table 3 and Figure 1a) compared to non-athletes (FWE-corrected, *p* < 0.05).

### 3.3. Resting-State Function Activity Alterations

The results showed that athletes had higher ALFF values in the right cerebellar Crus I region (Table 4 and Figure 1b). The ReHo values in the right cerebellar Crus II to lobule VII b area were higher in the athlete group than in the control group (Table 4 and Figure 1c). Differences in DC and fALFF between the two groups were not significant.

### 3.4. Microstructure Alterations

**Figure 2:**
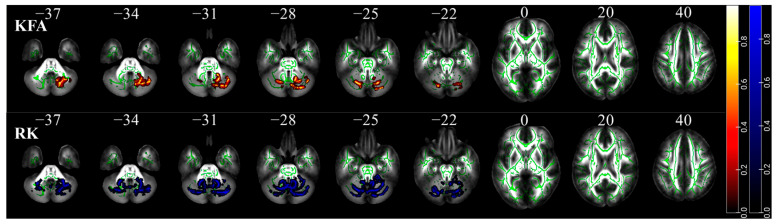
TBSS results indicated that KFA was higher and RK was lower in basketball athletes than non-athletes. Permutation test with 5000 iterations; *p* < 0.05 TFCE correction. The skeleton mask is shown in green. Areas of RK reduction are shown in blue. Areas of increased KFA are shown in red.

We observed significant changes in the DKI indices between the two groups, including a significant increase in KFA values and a significant decrease in RK values only in the cerebellum. Based on a Cerebellar White Matter Atlas (CWMA, white matter atlas) [34] and the SUIT template (gray matter atlas), our results indicate that athletes showed decreased RK values in the bilateral middle cerebellar peduncle, bilateral inferior cerebellar peduncle, bilateral Crus I, bilateral lobule VI, right Crus II, and right lobule V (Table 5 and Figure 2 for details). Concurrently, there was an increase in KFA values in almost the same regions, including the right middle cerebellar peduncle, right inferior cerebellar peduncle, bilateral cerebellar Crus I, bilateral lateral lobule VI, and right cerebellar Crus II (Table 5 and Figure 2). There were no significant differences in DTI parameters, MK, or RK between the two groups.

### 3.5. Correlation Between Training Experience and Physical Abilities

**Figure 3:**
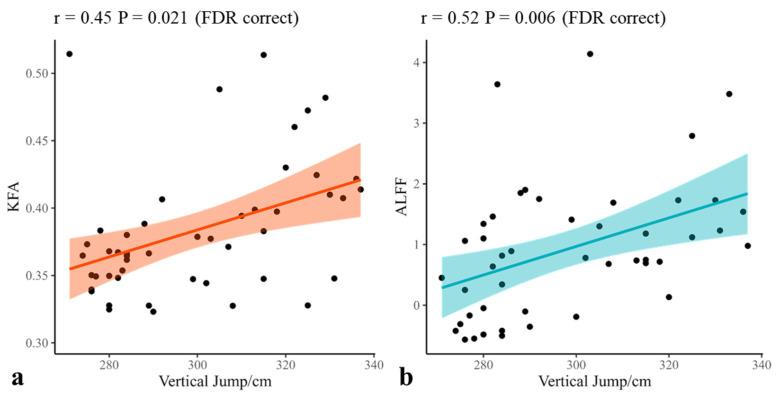
Individual KFA/ALFF values for the surviving clusters of basketball athletes were extracted for Spearman correlation with physical abilities. (**a**) The KFA value in the cerebellum was positively correlated with the vertical jump score. (**b**) The ALFF value in the cerebellar Crus I was positively correlated with the vertical jump scores. KFA: kurtosis fractional anisotropy, ALFF: Amplitude of Low-Frequency Fluctuations.

The correlations between specific brain measures and physical performance in basketball athletes were assessed using Spearman correlation analysis (Supplementary Table S3 for all results). Specifically, there was a positive correlation between the KFA value in the cerebellum and vertical jump score (r = 0.45, *p* = 0.021 FDR corrected, Figure 3). Additionally, the ALFF value in cerebellar Crus I showed positive correlations with vertical jump score (r = 0.52, *p* = 0.006, FDR-corrected, Figure 3). Furthermore, the ReHo value in the right cerebellar Crus II to lobule VII b area in basketball athletes was positively correlated with years of sport training (r = 0.30, *p* = 0.037 uncorrected) and years of basketball training (r = 0.31, *p* = 0.032 uncorrected). Lastly, no correlations were found between the RK within significantly altered brain regions and the basketball athletes’ training experience and physical abilities. Pearson correlation results were also presented in Table S4.

## 4. Discussion

This study examined cerebellar adaptations in basketball athletes using multimodal MRI. Compared with matched non-athletes, basketball athletes exhibited (i) greater GMV in the cerebellar Crus I, (ii) extensive microstructural alterations in both cerebellar cortex and peduncles, and (iii) enhanced functional activity in the Crus I/II regions. Significantly, some of these indices were positively correlated with vertical jump performance, a core basketball-specific motor ability. These findings suggest that basketball athletes exhibit multidimensional cerebellar enhancement and that such adaptations may partly be linked to their athletic performance.

In basketball athletes, gray matter enlargement was primarily observed in the cerebellar Crus I. An increase in gray matter volume usually indicates better functional performance. Functionally, Crus I has been consistently identified as a core hub of the cerebellum [35], mainly engaged in both higher-order cognitive processes [1,36] and motor functions [37]. The structural changes in this region may bring a faster and more effective cerebellum internal model for athletes and results in better motor and cognition performance. This inference aligns well with the integrated cognitive–motor demands of basketball, where athletes must perform precise movements while making rapid tactical decisions [20]. Such structural enhancement may therefore be functionally meaningful for athletes’ optimizing performance in highly dynamic game contexts.

Functional alterations were also observed in Crus I and Crus II. The increase in the ALFF signal in Crus I converges with the structural findings, reinforcing the idea that this region may represent a consistent site of cerebellar adaptation in basketball athletes. Crus II, functionally adjacent to Crus I, is similarly involved in higher-order cognitive processes that are closely linked to motor functions [1,38]. Enhanced regional coherence is associated with faster local information processes. The functional enhancement of this region may allow athlete form superior cerebellar function, thereby supporting the efficient integration of motor and cognitive performance [39,40]. The functional findings were highly consistent with the structural results. Cerebellar adaptations in athletes seemed to be themed on motor-cognitive integration, which directly mirrors the core requirements of basketball.

DKI analysis revealed extensive microstructural alterations in basketball athletes. At the white matter level, increased KFA and decreased RK generally indicate more ordered and efficient neural fiber structures [41,42,43]. These changes were predominantly located in the cerebellar peduncles, which constitute the only pathways through which the cerebellum communicates with other brain regions [44]. This finding suggests that the structural connectivity between the cerebellum and the broader brain network may be enhanced in basketball athletes. This enhancement provides an opportunity for athletes to perform a faster and more extensive information exchange between cerebellum and cerebral cortex. Such structural remodeling may underlie our previous observation of an enhanced cerebellar connection to relevant cerebral cortical areas during cognitive tasks [2,45].

At the cortical level, increased KFA and decreased RK imply the emergence of novel oriented microstructures [41,42,43], potentially reflecting processes such as the expansion of parallel fibers that enable faster and more efficient information processing. These cortical alterations were also concentrated around Crus I and lobule VI. Notably, lobule VI belongs to both motor and cognitive representations of the cerebellum [38], whose functions are highly relevant to the demands of basketball. The recurrent involvement of Crus I across structural, microstructural, and functional analyses further supports the notion that it is a core locus of adaptive changes in the cerebellum of basketball athletes.

A key finding of this study was that cerebellar KFA and ALFF values in basketball athletes were positively correlated with vertical jump performance—a sport-specific skill reflecting lower-limb power and intermuscular coordination. Although these associations were of moderate strength, this result is consistent with prior studies that suggest a potential link between physical abilities and improved neurophysiological function [21,46]. From a neural perspective, vertical jump performance may be modulated by cerebellar–cortical circuits regulation [6], whose functional activity has been associated with not only motor performance [3], but also with cognitive functions [39]. This raises the possibility that adaptive cerebellar changes could be related not only to motor performance (e.g., vertical jump) but also to cognitive abilities (e.g., in-game decision-making). Alternatively, these relationships may reflect broader improvements in neuromuscular efficiency or coordination rather than direct cerebellar modulation of power output. The correlations were not found in general physical fitness and training indexes, implying that cerebellar adaptations are likely more closely related to sport-specific demands than to general fitness or training duration, although this interpretation should be viewed with caution. Taken together, these findings tentatively suggest that cerebellar adaptations may contribute to the refinement of sport-specific skills. It will be important for future work to directly investigate the link between these cerebellar changes and specific task performance using longitudinal or task-based designs to better clarify their causal and functional significance.

Overall, basketball athletes demonstrated widespread cerebellar adaptations, encompassing structural, microstructural, and functional levels. These changes were centered on the Crus I, which not only modulates motor control but is also consistently recruited during cognitive tasks. Crus I may represent a consistent site of adaptation of cerebellar adaptations in basketball athletes, contributing to athletes’ advantages in both motor execution. While our behavioral assessments were limited to motor outcomes, it is plausible that cerebellar adaptations extend to cognitive domains such as working memory and decision-making, which are equally critical in basketball. Future studies incorporating cognitive tasks will be necessary to further test this possibility.

Several limitations warrant consideration. First, while DKI parameters suggest microstructural reorganization, the biological substrates of these changes (e.g., synaptic density vs. glial alterations) remain speculative and necessitate histopathological validation. Second, the cross-sectional design precludes causal inferences regarding training-induced plasticity; longitudinal or training-intervention designs that could track cerebellar plasticity over time are needed. Third, the relationship between cerebellar adaptation and specific sport performance remains incompletely understood. The functional implications of observed adaptations should be further tested using task-based fMRI paradigms and standardized cognitive-motor assessments to assess whether the same cerebellar regions support basketball-specific decision-making, not only physical performance. Lastly, a larger sample size is necessary to further validate the results of this study and compare them with other sports programs.

## 5. Conclusions

This multimodal study provides the first evidence that long-trained basketball athlete performed coordinated neural remodeling in the cerebellum, especially in Crus I. These findings not only delineate a map of cerebellar adaptation underlying exceptional athletic performance but, more profoundly, corroborate the cerebellum’s functional role as a hub for cognitive-motor integration. This work offers novel experimental insights and a theoretical framework for understanding experience-dependent plasticity in the human brain. These adaptations likely underpin the superior sensorimotor coordination, split-second decision-making, and kinematic precision demanded by elite basketball performance. Future work should explore whether cerebellar biomarkers could guide talent development or injury rehabilitation in sports medicine.

## Figures and Tables

**Table 1:** Demographics and physical abilities of both groups.

	Basketball Athletes	Non-Athletes	*p*
Gender (F, M)	25, 30	26, 29	0.847
Age (M ± SD)	20.75 ± 1.83	20.87 ± 1.98	0.727
Educated Years	14.75 ± 1.82	14.80 ± 1.67	0.870
Years of Sport Training	9.25 ± 3.22	-	-
Years of Basketball Training	8.44 ± 2.69	-	-
Training time (hours/week)	7.21 ± 4.50	-	-
Vertical Jump (cm)	299.29 ± 23.36	-	-
Dribbling (s)	39.93 ± 2.19	-	-
Shooting percentage (%)	0.68 ± 0.14	-	-
Agility (s)	16.74 ± 2.47	24.77 ± 5.92	<0.001
Gait Speed (m/s)	1.52 ± 0.44	1.51 ± 0.12	0.182
Explosive power (cm)	33.79 ± 7.39	33.04 ± 8.43	0.704

**Table 2:** MRI scanning parameters.

Imaging Methods	Geometry Parameters	Contrast Parameters
T1-weighted imaging	FOV = 256 mm × 256 mm Slice number = 192 Voxel size = 1 × 1 × 1 mm^3^	TR = 2530 ms TE = 2.98 ms TI = 1100 ms flip angle = 7°
Diffusion-weighted imaging (DWI)	FOV = 224 mm × 224 mm Slice number = 74 Voxel size = 2 × 2 × 2 mm^3^	TR = 5000 ms TE = 95 ms b = 0, 1000, 2000 s/mm^2^ directions = 30 flip angle = 90°
BOLD weighted imaging	FOV = 192 mm × 192 mm Slice number = 58 interslice gap = 20% Voxel size = 2 × 2 × 2 mm^3^	TR = 2000 ms, TE = 30 ms, flip angle = 90°
Field map for DWI	FOV = 224 mm × 224 mm Slice number = 74 Voxel size = 2 × 2 × 2 mm^3^	TR = 735 ms, TE = 4.92 & 7.38 ms flip angle = 90°
Field map for fMRI	FOV = 192 mm × 192 mm Slice number = 58 interslice gap = 20% Voxel size = 2 × 2 × 2 mm^3^	TR = 571 ms, TE = 4.92 & 7.38 ms flip angle = 90°

FOV: field of view, TR: repetition time, TE: echo time, TI: inversion time.

**Table 3:** GMV differences between basketball athletes and non-athletes.

Cluster	Area	Size	MNI (mm)			Peak T	*p*
GMV 1	Left Crus I	79	−23	−73	−35	4.21	0.023
GMV 2	Right Crus I	113	28	−73	−33	3.99	0.012

GMV: gray matter volume.

**Table 4:** Resting-state functional brain activity differences between basketball athletes and non-athletes.

Cluster	Area	Size	MNI (mm)			Peak T	*p*
ALFF	Right Crus I	48	36	−80	−31	3.87	0.002
ReHo	Right Crus II and right VII b	58	22	−68	−41	3.65	0.038

ALFF: Amplitude of Low-Frequency Fluctuations, ReHo: Regional Homogeneity.

**Table 5:** DKI metrics difference between basketball athletes and non-athletes.

Cluster	Area	Size	MNI (mm)			Peak T	*p*
RK		4758	12	−53	−25	5.09	0.023
	White matter (CWMA)						
	Right Middle Cerebellar Peduncle	592					
	Right Inferior Cerebellar Peduncle	512					
	Left Inferior Cerebellar Peduncle	424					
	Left Middle Cerebellar Peduncle	320					
	Gray matter (SUIT)						
	Right Crus I	746					
	Right VI	452					
	Left VI	419					
	Left Crus I	301					
	Right Crus II	174					
	Right V	127					
	Right IX	94					
	Vermis VI	86					
	Vermis IX	53					
KFA		1378	33	−59	−43	3.86	0.031
	White matter (CWMA)						
	Right Middle Cerebellar Peduncle	389					
	Right Inferior Cerebellar Peduncle	154					
	Gray matter (SUIT)						
	Right Crus I	249					
	Right VI	151					
	Left VI	140					
	Left Crus I	101					
	Right Crus II	58					

DKI: Diffusion kurtosis Image, KFA: kurtosis fractional anisotropy, RK: radial kurtosis; CWMA: white matter atlas, SUIT: gay matter atlas.

## Data Availability

The datasets generated during and/or analyzed during the current study are available from the corresponding author upon reasonable request.

## Supplementary Materials

The following supporting information can be downloaded at: https://www.mdpi.com/article/10.3390/brainsci15111221/s1, Table S1: Physical fitness test method; Table S2: The definitions of diffusion measures; Table S3: All results of correlation analysis; Table S4: All results of partial correlation analysis; Figure S1: Dribbling test diagram.

## Abbreviations

The following abbreviations are used in this manuscript:

AD	axial diffusivity
AK	axial kurtosis
ALFF	Amplitude of Low-Frequency Fluctuations
DC	Degree Centrality
DKI	Diffusion Kurtosis Imaging
DTI	Diffusion Tensor Imaging
FA	fractional anisotropy
fALFF	fractional ALFF
GMV	gray matter volume
KFA	kurtosis fractional anisotropy
MD	mean diffusivity
MK	mean kurtosis
MRI	Magnetic Resonance Imaging
RD	radial diffusivity
ReHo	Regional Homogeneity
RK	radial kurtosis
TBSS	Tract-Based Spatial Statistics
TFCE	Threshold-Free Cluster Enhancement
