# Supplementary material for: Adaptations in the Structure and Function of the Cerebellum in Basketball Athletes
Source: Brain Sci. 2025 Nov 13;15(11):1221. doi: 10.3390/brainsci15111221 (PMC12650634; doi:10.3390/brainsci15111221)
Supplement: Supplementary file 1 [file brainsci-15-01221-s001.zip › brainsci-3951566-supplementary.pdf]

Table S1. Physical fitness test method.

Name	Test methods	Unit
**Agility**	The agility test was conducted using the hexagon test. Before the test, a regular hexagon with a side length of 60 centimeters was marked on the ground using tape. A cross was drawn at the center of the hexagon to indicate the starting and returning position. At the beginning of the test, the subject stood at the center of the hexagon, facing any of its sides. Following a clockwise direction, they jumped outside each side of the hexagon and then returned to the center point. This jumping out and returning sequence was repeated until three full rounds were completed, considered one trial.<br>Timing for the test commenced when the subject first left the center point and ended when they returned to the center point for the final time. Each time a subject stepped on the boundary line or failed to return to the center point, it was considered an error, adding 0.5 seconds to the total time for each error. The time taken to complete the test was recorded as the agility score.	s
**Gait Speed**	The participants were instructed to walk a short distance of 4 meters at their normal pace. However, they had to cover a longer distance of 6 meters as we recorded their time 2 meters after they began walking. The test was conducted twice, and the time taken to cover the 4-meter distance was recorded in seconds. The faster of the two trials was used for scoring. The scores were then calculated as meters per second.	m/s
**Explosive power**	The Countermovement Jump (CMJ) test is a way to measure the explosive power of the lower limbs. In this test, the subject stands on a force plate (Kistler, 9281EA, SWE) with their hands on their hips in a natural standing position. When they hear the start command, the subject jumps upward with maximum effort, keeping their body straight and their knees extended before landing again. The plate automatically displays the height at which the participant has just jumped. The test is then repeated after a 30-second rest period, and this is done three times, with the best score being recorded.	cm
**Vertical Jump**	We attached stickers with height markers on a vertical wall. The subject stood naturally with feet apart in front of the wall. They could crouch or swing their arms to increase their jump height before jumping and using one hand to touch the highest possible point on the wall. The height reached by the subject's fingertips was recorded as the jump height. The test was repeated three times, and the best result was recorded as the final score.	cm
**Shooting percentage**	During a basketball shooting test, the participant was asked to stand at the free throw line to take free throws. To calculate the shooting percentage, the number of shots made is divided by the total number of shots taken. In this particular test, the participant will take 20 shots.	%
**Dribbling**	Six obstacles were set up on the court (see Figure S1). At the start of the test, the subject dribbled quickly from point A to obstacle 1, performed a front crossover, then dribbled to obstacle 2 and executed a between-the-legs dribble. The subject continued to obstacle 3, performed a spin move, and made a layup. After scoring, they retrieved the rebound and dribbled to Obstacle 4, performing a behind-the-back dribble, then dribbled to Obstacle 5 for another between-the-legs dribble. Finally, they dribbled to obstacle 6, performed another spin	s

	move, and made a second layup. After scoring, they retrieved the rebound and repeated the sequence in reverse.<br>During the test, violations such as double dribbling, traveling, missing a move, or not reaching the designated areas (being too far from obstacles) were penalized by adding 2 seconds for each occurrence. Three testers conducted the timing simultaneously, mainly to detect violations like stepping on the lines. The total time to complete the course was recorded as the final score.

Table S2. The definitions of diffusion measures.

Measures	Definitions
**DTI parameters**	
**AD**	The axial diffusivity. The formula is as follows:<br>$AD = \lambda_1$<br>Where λ_1 is the maximum eigenvalue of tensor matrix D.<br>$\boldsymbol{D} = \begin{bmatrix} D_{xx} & D_{xy} & D_{xz} \\ D_{yx} & D_{yy} & D_{yz} \\ D_{zx} & D_{zy} & D_{zz} \end{bmatrix}$
**RD**	The radial diffusivity. The formula is as follows:<br>$RD = \frac{\lambda_2 + \lambda_3}{2}$<br>Where$\lambda_2$, λ_3 are the other two eigenvalues of the tensor matrix D.
**MD**	The mean diffusivity. The formula is as follows:<br>$MD = \frac{\lambda_1 + \lambda_2 + \lambda_3}{3}$
**FA**	The fractional anisotropy. The formula is as follows:<br>$FA = \sqrt{\frac{(\lambda_1 - MD)^2 + (\lambda_2 - MD)^2 + (\lambda_3 - MD)^2}{2(\lambda_1^2 + \lambda_2^2 + \lambda_3^2)}}$
**DKI parameters**	
**MK**	The mean kurtosis. The formula is as follows:<br>$MK = \frac{1}{n}\sum_{i=1}^{n}(K_{app})_i$<br>Where K_{app} is the apparent diffusion kurtosis:<br>$K_{app} = \frac{MD^2}{D_{app}^2}\sum_{i=1}^{3}\sum_{j=1}^{3}\sum_{k=1}^{3}\sum_{l=1}^{3}e_i e_j e_k e_l W_{ijkl}$<br>Where D_{app} is apparent diffusion coefficient (i.e., ADC), and W is 4th order diffusion kurtosis tensor matrix.
**AK**	The axial kurtosis. The formula is as follows:<br>$AK = K_1$<br>where<br>$K_i = \frac{MD^2}{\lambda_i^2}\widehat{W}_{iiii}$<br>Where<br>$\widehat{W}_{ijkl} = \sum_{i'=1}^{3}\sum_{j'=1}^{3}\sum_{k'=1}^{3}\sum_{l'=1}^{3}e_{i'i}e_{j'j}e_{k'k}e_{l'l}W_{i'j'k'l'}$
**RK**	The radial kurtosis. The formula is as follows:<br>$RK = \frac{K_2 + K_3}{2}$

**KFA**	Kurtosis Fractional Anisotropy. The formula is as follows: $$KFA = \sqrt{\frac{3}{2}\frac{(K_1 - \bar{k})^2 + (K_2 - \bar{k})^2 + (K_3 - \bar{k})^2}{k_1^2 + k_2^2 + k_3^2}}$$

Table S3. All results of Spearman correlation analysis.

Pairs	r	p	pfdr
**RK-Age**	–0.02	0.877	0.924
**RK-EY**	–0.04	0.777	0.897
**RK-YST**	–0.00	0.997	0.997
**RK-YBT**	–0.09	0.531	0.776
**RK-Agility**	0.15	0.409	0.701
**RK-GS**	–0.06	0.751	0.892
**RK-EP**	0.03	0.886	0.925
**RK-Dribbling**	0.15	0.474	0.736
**RK-VG**	–0.20	0.19	0.468
**RK-SP**	–0.04	0.837	0.905
**RK-TT**	-0.08	0.596	0.796
**KFA-Age**	–0.00	0.984	0.997
**KFA-EY**	–0.01	0.967	0.992
**KFA-YST**	–0.09	0.518	0.768
**KFA-YBT**	–0.03	0.835	0.905
**KFA-Agility**	–0.16	0.363	0.678
**KFA-GS**	0.15	0.392	0.698
**KFA-EP**	0.22	0.219	0.494
**KFA-Dribbling**	–0.23	0.261	0.542
**KFA-VG**	0.45	0.002	0.021
**KFA-SP**	0.23	0.262	0.542
**KFA-TT**	0.20	0.165	0.469
**GMV-L-Age**	–0.22	0.103	0.315
**GMV-L-EY**	–0.08	0.567	0.791
**GMV-L-YST**	0.03	0.813	0.903
**GMV-L-YBT**	–0.13	0.346	0.669
**GMV-L-Agility**	0.22	0.207	0.489
**GMV-L-GS**	–0.10	0.582	0.795
**GMV-L-EP**	0.26	0.135	0.376
**GMV-L-Dribbling**	0.19	0.343	0.669
**GMV-L-VG**	0.04	0.800	0.900
**GMV-L-SP**	0.25	0.219	0.494
**GMV-L-TT**	0.13	0.345	0.686
**GMV-R-Age**	–0.19	0.182	0.218
**GMV-R-EY**	–0.12	0.398	0.698
**GMV-R-YST**	0.10	0.46	0.736
**GMV-R-YBT**	–0.05	0.733	0.887
**GMV-R-Agility**	0.09	0.603	0.795
**GMV-R-GS**	–0.14	0.431	0.729
**GMV-R-EP**	0.15	0.401	0.698

**GMV-R-Dribbling**	0.30	0.143	0.39
**GMV-R-VG**	0.13	0.371	0.678
**GMV-R-SP**	0.08	0.706	0.883
**GMV-R-TT**	0.13	0.349	0.686
**ReHo-Age**	–0.11	0.45	0.734
**ReHo-EY**	0.04	0.802	0.9
**ReHo-YST**	0.30	0.037	0.202
**ReHo-YBT**	0.31	0.032	0.202
**ReHo-Agility**	–0.05	0.767	0.894
**ReHo-GS**	–0.35	0.046	0.218
**ReHo-EP**	–0.27	0.123	0.352
**ReHo-Dribbling**	0.25	0.23	0.494
**ReHo-VG**	–0.25	0.098	0.315
**ReHo-SP**	0.10	0.642	0.829
**ReHo-TT**	-0.01	0.933	0.961
**ALFF-Age**	0.05	0.739	0.887
**ALFF-EY**	0.03	0.862	0.915
**ALFF-YST**	–0.19	0.181	0.463
**ALFF-YBT**	–0.26	0.167	0.219
**ALFF-Agility**	–0.04	0.822	0.905
**ALFF-GS**	0.32	0.066	0.248
**ALFF-EP**	0.33	0.060	0.239
**ALFF-Dribbling**	0.00	0.988	0.997
**ALFF-VG**	0.52	< .001	0.006
**ALFF-SP**	–0.12	0.561	0.791
**ALFF-TT**	0.12	0.402	0.700

EY: Educated Years; YST: Years of Sport Training; YBT: Years of Basketball Training; VJ: Vertical Jump; SP: Shooting percentage; GS: Gait Speed; EP: Explosive power; TT: Training Time

* p < 0.05; ** p < 0.01

Table S4. All results of partial correlation analysis.

Pairs	r	P	P(FDR)
KFA-YST	-0.14	0.335	0.439
KFA-YBT	-0.08	0.562	0.632
KFA-TT	0.14	0.342	0.439
KFA-PS	0.24	0.274	0.439
KFA-Dribbling	-0.22	0.316	0.439
KFA-VJ	0.46	**0.002****	**0.016***
KFA-Agility	-0.05	0.777	0.777
KFA-EP	0.28	0.132	0.439
KFA-GS	0.25	0.178	0.439
RK-YST	0.09	0.556	0.998
RK-YBT	-0.01	0.924	0.998
RK-TT	-0.11	0.467	0.998
RK-PS	-0.09	0.676	0.998

Pairs	r	P	P(FDR)
RK-Dribbling	0.21	0.346	0.998
RK-VJ	-0.21	0.177	0.998
RK-Agility	0.04	0.842	0.998
RK-EP	0.00	0.998	0.998
RK-GS	-0.17	0.357	0.998
ReHo-YST	0.25	0.092	0.277
ReHo-YBT	0.30	**0.041***	0.186
ReHo-TT	-0.16	0.272	0.423
ReHo-PS	-0.01	0.962	0.962
ReHo-Dribbling	-0.01	0.952	0.962
ReHo-VJ	-0.16	0.304	0.423
ReHo-Agility	-0.18	0.329	0.423
ReHo-EP	-0.21	0.258	0.423
ReHo-GS	-0.43	**0.015***	0.132
ALFF-YST	-0.21	0.155	0.279
ALFF-YBT	-0.26	0.089	0.126
ALFF-TT	0.04	0.795	0.894
ALFF-PS	-0.14	0.535	0.713
ALFF-Dribbling	0.02	0.917	0.917
ALFF-VJ	0.49	**<0.001*** **	**0.008** **
ALFF-Agility	-0.11	0.555	0.713
ALFF-EP	0.37	**0.040***	0.126
ALFF-GS	0.31	0.094	0.211
VBM-R-YST	0.14	0.310	0.851
VBM-R-YBT	-0.04	0.756	0.851
VBM-R-TT	0.08	0.582	0.851
VBM-R-PS	0.08	0.723	0.851
VBM-R-Dribbling	0.19	0.366	0.851
VBM-R-VJ	0.08	0.588	0.851
VBM-R-Agility	0.11	0.563	0.851
VBM-R-EP	0.00	0.987	0.987
VBM-R-GS	-0.17	0.347	0.851
VBM-L-YST	-0.02	0.880	0.880
VBM-L-YBT	-0.18	0.213	0.497
VBM-L-TT	0.16	0.270	0.497
VBM-L-PS	0.32	0.126	0.497
VBM-L-Dribbling	0.06	0.780	0.878
VBM-L-VJ	0.07	0.641	0.824
VBM-L-Agility	0.24	0.192	0.497
VBM-L-EP	0.20	0.276	0.497
VBM-L-GS	-0.09	0.610	0.824

EY: Educated Years; YST: Years of Sport Training; YBT: Years of Basketball Training; VJ: Vertical Jump; SP: Shooting percentage; GS:

Gait Speed; EP: Explosive power; TT: Training time; * $p < 0.05$; ** $p < 0.01$; *** $p < 0.001$

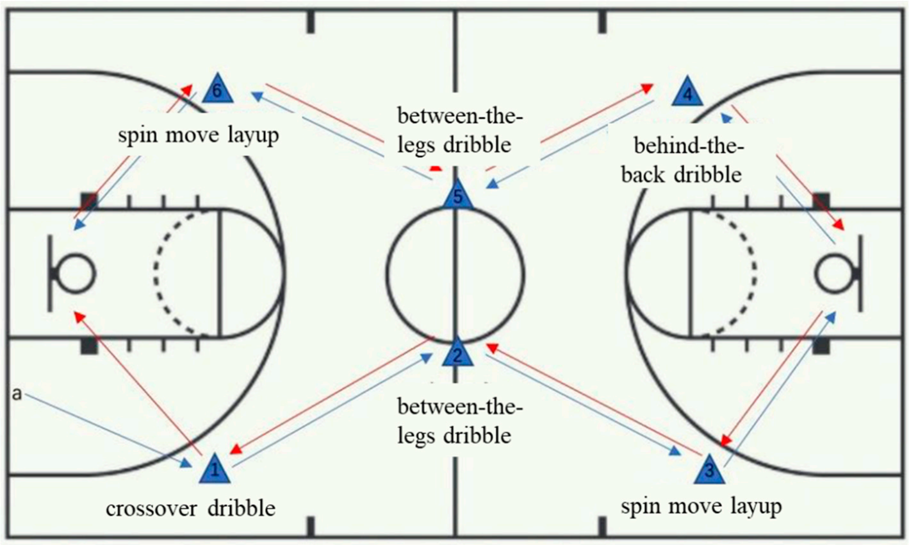

Figure S1 Dribbling test diagram.
